# How did Canadian national team athletes manage the COVID-19 pandemic? Athlete, coach, and support staff perspectives to guide future responses to major stressors

**DOI:** 10.3389/fpsyg.2025.1540372

**Published:** 2025-07-23

**Authors:** Lori Dithurbide, Véronique Boudreault, Natalie Durand-Bush, Gabriel Delage, Lucy MacLeod, Véronique Gauthier

**Affiliations:** ^1^School of Health and Performance, Dalhousie University, Halifax, NS, Canada; ^2^Faculté des sciences de l’activité physique, University of Sherbrooke, Sherbrooke, QC, Canada; ^3^School of Human Kinetics, University of Ottawa, Ottawa, ON, Canada; ^4^Département de psychologie, Université du Québec à Trois-Rivières, Trois-Rivières, QC, Canada

**Keywords:** coronavirus, sport, high-performance, sport psychology, well-being

## Abstract

As we move beyond the pandemic and the lockdowns it imposed on elite athletes, there remains much to learn from how they adapted to this unprecedented crisis. Given the significant impact on their mental health—and the likelihood that they will face other major stressors throughout their careers—this study explored the interplay between the COVID-19 pandemic, mental health, and mental performance among Canadian national team athletes. The perspectives of athletes, coaches, and support staff were examined through focus groups and interviews conducted during the second wave of the pandemic in Canada. Participants included 25 athletes, eight coaches, and five support staff members. Inductive reflexive thematic analysis generated three main themes: (a) consequences of COVID-19 for athletes, (b) factors influencing athlete mental health, and (c) coach and support staff perspectives on well-being and evolving roles. Athletes reported a range of experiences influenced by factors such as isolation, stigma, coping skills, and social support. Mental health and mental performance emerged as core components of a culture of excellence—interrelated and mutually reinforcing. These findings underscore the importance of fostering environments that prioritize well-being alongside performance, particularly as sport organizations prepare for future periods of uncertainty and stress.

## Introduction

The wellbeing of national team athletes has been affected by the COVID-19 pandemic (Dithurbide et al., 2022; Haan et al., 2021; Uroh and Adewunmi, 2021). While we are moving away from the pandemic and the lockdowns it generated in high performance sport, there is still a lot to learn from athletes’ adaptation to this stressful crisis. Athletes may face challenges like the ones they experienced during the pandemic for example, disruption of their normal routine, being prevented from practicing their sport, uncertainty about their possibility to perform, stress, and lack of perceived control in their life. Notably, various forms of loss or disruption, such as bereavement or season-ending injuries, have been shown to affect athletes in similar ways as the COVID-19 pandemic (Fogaca et al., 2021; Moore et al., 2021; Simpson and Elberty, 2018). At the national level, athletes are expected to perform at their best in international competitions, which requires intensive training, preparation, and dedication despite potential unexpected challenges or setbacks. Therefore, it is valuable to understand how the pandemic has affected national team athletes’ mental health and mental performance, as these are key factors for their long-term well-being and success in sport.

Mental health is defined as “a state of well-being in which every individual realizes his or her own potential, can cope with the normal stresses of life, can work productively and fruitfully, and is able to make a contribution to her or his community” (World Health Organization, 2014). Mental health is influenced by various biological, psychological, social, and environmental factors that interact in complex ways (World Health Organization, 2014). In sports, mental health can be positively or negatively affected depending on how athletes perceive and manage these factors (Henriksen et al., 2020). For example, sport participation can enhance mood, reduce stress, improve cognitive function, and prevent depression (Gustafsson et al., 2016). However, at competitive elite level, it can also lead to overtraining syndrome, burnout, or mental illness like eating disorders if done excessively or compulsively (Reardon et al., 2019). Similarly, social support can provide emotional comfort, instrumental assistance, informational guidance (e.g., feedback from coaches about performance), and motivational encouragement for athletes, but it can also create pressure, conflict, or isolation if it is inadequate or inappropriate (Freeman, 2021; Rees and Hardy, 2000). Therefore, it is essential for athletes to balance the demands and rewards of sport participation and to develop effective mental skills to deal with stressors that may affect their mental health (Biddle and Asare, 2011).

Mental performance is defined as “the psychological processes that enable an individual to perform at an optimal level in a given situation” (Weinberg and Gould, 2015, p. 3). Mental performance is influenced by various psychological skills (i.e., mental performance skills) that can be learned and trained such as attention, concentration, self-talk, imagery, relaxation, arousal regulation, goal-setting, self-confidence, motivation, emotion management, and coping (Durand-Bush et al., 2023). These skills help athletes to focus on relevant cues, regulate their thoughts and emotions, enhance their self-belief and motivation, and overcome challenges and setbacks in sport (Durand-Bush et al., 2023). Mental performance is also influenced by various psychological factors that can be modified or adapted such as personality, attitudes, beliefs, values, expectations, and attributions (Weinberg and Gould, 2015). These factors shape how athletes perceive and interpret their sport experiences and how they respond to different situations and outcomes in sport (Weinberg and Gould, 2015). Therefore, it is important for athletes to develop and maintain mental performance skills that enable them to perform at their best in sport (Durand-Bush et al., 2023; Weinberg and Gould, 2015).

Life events such as bereavement, injuries and other isolating situations have been reported to have an impact on athlete’s mental health and well-being (Fogaca et al., 2021; Moore et al., 2021; Simpson and Elberty, 2018). Specifically, the COVID-19 pandemic is one life event that has had a significant impact on both the mental health and mental performance of athletes. Several studies have reported that athletes have experienced increased levels of stress, anxiety, depression, anger, boredom, loneliness, and grief during the pandemic (e.g., Brand et al., 2020; Costa et al., 2020; Davis et al., 2020; Schinke et al., 2020). These negative emotions have been associated with various sources of stress, such as the lack of training opportunities, the uncertainty about the future of sport, the loss of goals and competitions, the disruption of social support networks, the fear of infection or contagion, the financial difficulties, and the personal or family issues related to the pandemic (e.g., Boudreault et al., 2022; Brand et al., 2020; Costa et al., 2020; Davis et al., 2020; Schinke et al., 2020). These stressors have also affected the mental performance of athletes, as they have reduced their motivation, confidence, concentration, and enjoyment in sport (e.g., Brand et al., 2020; Costa et al., 2020; Davis et al., 2020; Schinke et al., 2020). Moreover, some athletes have reported difficulties in maintaining or developing their mental performance skills during the pandemic, such as goal setting, imagery, self-talk, relaxation, and coping (e.g., Brand et al., 2020; Costa et al., 2020; Davis et al., 2020; Schinke et al., 2020).

However, not all athletes have experienced the pandemic in the same way. Some athletes have reported positive effects of the pandemic on their mental health and mental performance, such as increased resilience, optimism, gratitude, creativity, flexibility, and growth (e.g., Davis et al., 2020; Schinke et al., 2020). These positive outcomes have been attributed to various factors that have helped athletes to cope with the pandemic, such as social support, physical activity, communication, mindfulness, self-care, humor, hobbies, and spirituality (e.g., Dithurbide et al., 2022). These factors have also helped athletes to enhance or maintain their mental performance skills during the pandemic, such as attention, concentration, self-confidence, motivation, emotion regulation, and coping (e.g., Davis et al., 2020; Dithurbide et al., 2022; Schinke et al., 2020). Moreover, some athletes have used the pandemic as an opportunity to work on their mental performance skills or to learn new skills that could benefit their sport performance in the future (e.g., Davis et al., 2020; Dithurbide et al., 2022; Schinke et al., 2020). While such findings have enhanced our understanding of how elite athletes may cope during large-scale disruptions, much of the existing literature is limited to athlete self-report (e.g., Boudreault et al., 2022) or to the perspectives of practitioners, such as mental performance consultants or psychologists working with this population (Dithurbide et al., 2022).

The role of coaches and support staff in supporting athletes’ mental health and mental performance during the pandemic has been highlighted by several studies (e.g., Bissett et al., 2020; Henriksen et al., 2020; Poucher et al., 2023). Coaches and support staff are key agents of social support for athletes, as they provide emotional, instrumental, informational, and motivational support (Rees and Hardy, 2000). Coaches and support staff can also influence athletes’ mental health and mental performance by creating a positive and supportive environment, by fostering a sense of autonomy, competence, and relatedness among athletes, by setting realistic and flexible goals, by providing constructive feedback, by facilitating communication and collaboration, by promoting mental performance skills, and by addressing any psychological issues or concerns that may arise during challenging situation such as the pandemic (e.g., Brand et al., 2020; Purcell et al., 2022). Moreover, a coach’s attitude and the importance they give to mental health has an important impact on an athlete’s own attitudes about their mental health (Poucher et al., 2023), adding to the importance of a coach’s role regarding their athlete’s mental health.

Recent advances in the field of mental health and mental performance in Canadian elite sport have led to innovative initiatives such as the Canadian Strategy for Mental Health in Sport (Durand-Bush and Van Slingerland, 2021) and the Gold Medal Profile for Sport Psychology (GMP-SP; Durand-Bush et al., 2023). These initiatives aim to promote flourishing mental health and develop mental performance skills for athletes, acknowledging that both aspects are interrelated and equally important for athletes’ well-being and excellence. Furthermore, they also recognize the importance of the ecological framework, which acknowledges the complex and layered relationships between the athlete and the systems surrounding them (i.e., microsystem, exosystem, and macrosystem; Purcell et al., 2019). The ecological framework acknowledges that the macrosystem [national/international sporting bodies (e.g., Sport Canada), the society and the media], the exosystem (athletes’ sport governing body) and the microsystem (coaches, teammates, family), play an important role in the athlete’s mental health, mental health literacy and mental health support. In coherence with the ecological framework, the importance given to appropriate mental performance support by each party of each system is closely related to the holistic view of ensuring an athlete’s overall wellbeing and adequate mental support to allow them to succeed and attain fulfillment in every aspect of their life. The framework also acknowledges that the closer one is to the athletes, the more direct the influence and responsibility become in prioritizing mental health and mental performance (Purcell et al., 2019). At Olympic and Paralympic level, coaches and support staff are not only part of the entourage, but also have authority and decision power that affect the athletes’ outcomes and satisfaction in all aspects of their life (Jowett and Cockerill, 2003; Turgeon et al., 2023). Therefore, they are central to the implementation of the Mental Health Strategy and the Gold Medal Profile. However, coaches and support staff may also face challenges in supporting athletes during the pandemic, such as adapting to new modes of delivery (e.g., online or remote), and dealing with their own mental health challenges. To this end, coaches and support staff are unique witnesses and first line supporters of athletes’ mental health and mental performance.

The current study was part of a larger program of research in which the perspective of mental health practitioners and mental performance consultants working with national team athletes was gathered examining the factors impacting athletes’ mental health, the consequences of COVID-19 on athletes’ mental health as well as the impact of the pandemic on their role (Dithurbide et al., 2022). Both mental health and mental performance practitioners perceived that athletes who had prior good mental performance skills coped better with the challenges related to the pandemic, suggesting the protective role of mental performance skills on the mental health of athletes. The data collected from practitioners closely collaborating with national team athletes revealed that, amid the pandemic, certain athletes encountered challenges, while others reported improved mental health due to reduced pressure and the advantages of having more personal time.

In order to further enhance our understanding of the complexity of the interplay between the COVID-19 pandemic and the mental health and mental performance of Canadian national team athletes, it is essential to gain insight from the athletes themselves as well as the coaches and support staff that surround them, allowing these parties to speak on how athletes can cope with and adapt to major disruptions in their sport careers. Therefore, the current study distinguishes itself from previous research by incorporating the perspectives of not only athletes, but also coaches and support staff on the topic of mental health and mental performance within the COVID-19 context. This novel contribution allows for a holistic understanding of the athlete’s experience of a life event, capturing the effect of interpersonal environments on mental health (Purcell et al., 2022).

The purpose of the current study was to examine the interplay between the COVID-19 pandemic, the mental health, and the mental performance of Canadian national team athletes. This research covered the perspective of athletes, but also that of coaches and support staff. A multiple perspectives qualitative approach was used to conduct the current study as it allows researchers to examine and understand the views of a group of individuals who have experienced the same event (i.e., COVID-19 pandemic; Santoro, 2014). While the focus was on athletes’ mental health and mental performance, gathering data from different individuals interacting with them in their daily training environment provided a more comprehensive account of their experiences and the factors that were impacting them (Santoro, 2014).

The specific objectives and research questions were:

- Objective 1: Examine the interplay between the COVID-19 pandemic and mental health.

1. How did athletes maintain their mental health during the pandemic and did their pre-pandemic mental health influence their responses to the pandemic and their ability to manage their mental health throughout?
2. What positively and negatively impacted athletes’ mental health during the pandemic?
3. What role did coaches and support staff play in supporting athletes’ mental health during the pandemic?

- Objective 2: Examine the interplay between the COVID-19 pandemic and mental performance.

1. How did athletes’ pre-pandemic mental performance skills influence their responses to the pandemic and their ability to self-regulate throughout?
2. How did athletes use the pandemic as an opportunity to work on their mental performance skills?
3. What role did coaches and support staff play in supporting athletes’ mental performance during the pandemic?

Data collection occurred between the months of December 2020 and March 2021, coinciding with Canada’s second wave of the COVID-19 pandemic. During this time, restrictions were still in place to prevent further spreading of the virus. Public health policies and regulations varied significantly depending on where participants lived and trained as each province within Canada acted independently. For example, some athletes were still isolated at home and trained on their own, where others were allowed to train with teammates at a training facility within a small group environment. Moreover, regulations within and outside of Canada evolved during the current study, which impacted both the participants and the sport communities in which they trained and competed.

## Methods

### Research team

The research team involved in the data collection and analysis included three researchers with academic positions at Canadian universities who are also Mental Performance Consultants (MPCs) with experience working in high-performance sport. These individuals have experience addressing both mental performance and mental health in their consulting practice and research. One is also a clinical psychologist. The team also included two graduate students who were involved in the data collection and analysis—one who has completed a research graduate degree in psychiatry and was a former Canadian national team athlete (i.e., sprint kayak) and the other who was completing a clinical psychology program and was a varsity student-athlete. The third graduate student on the research team supported the writing of the manuscript.

### Participants

The study included a total of 38 participants: 25 athletes (17 female, 8 male), eight coaches (two female, six male), and five support staff (three female, two male). Athletes represented 14 national sport organizations while coaches and staff represented 9 national sport organizations. Overall, there was representation from men and women, summer and winter, and Olympic and Paralympic sports. Athlete participants were required to be current Canadian national team athletes of an Olympic or Paralympic sport. Coaches and staff participants were required to work with at least one Canadian national team athlete or an Olympic or Paralympic sport team. Participant quotes in the Results are indicated as follows: A = Athlete, C = Coach, S = Support Staff.

### Procedure

Participants were recruited online through social media posts, including Instagram and Facebook. The digital study posters were shared by an account dedicated to the research project. In addition, the study posters were shared by relevant organizations (e.g., national training centers, national athlete support organizations). The participants were instructed to directly message or email the research team to inquire about participation. Participants were screened for eligibility and were then scheduled for an online focus group (athletes or coach/staff separately). Participants with minimal availability were scheduled for a virtual individual interview. The study included a total of 10 focus groups and 6 individual interviews. The focus group and individual interviews lasted an average of 75 and 40 min, respectively, and occurred in one of Canada’s official languages (i.e., English or French) following a predetermined, semi-structured question guide. The focus groups and interviews took place from December 2020 to February 2021, during Canada’s second wave of the COVID-19 pandemic for which public health restrictions varied across Canadian provinces.

### Data collection

Focus groups were selected as the main research method to explore the impact of COVID-19 on Canadian athletes. Focus groups foster dialogue and discussion among participants and allow the examination of both individual and collective perspectives in an interactive and efficient manner (Wilkinson, 1998). In a similar recent study, the research team collected meaningful data through focus groups and consolidated the perspectives of several mental performance and mental health service providers (Dithurbide et al., 2022). Two graduate students on the research team conducted the group and individual interviews in English or French based on participants’ main language.

The interview questions were designed to answer the research questions and encouraged dialogue amongst participants and researchers in a semi-structured manner (Rubin and Rubin, 2012). An example of a question is “What factors influenced your [athletes’] mental health during the pandemic and the ‘return to training’ phase?” The interview questions also explored how mental performance skills influenced athletes’ experiences and self-regulation during the pandemic (e.g., How did your mental performance skills influence your experience of the pandemic?).

Additionally, we asked participants about any resources that supported athletes, as well as gaps in mental health and mental performance resources. All participants were debriefed before and after the focus group and individual interviews to ensure their anonymity would be protected and to provide additional mental health resources. Participants received an CAD$20 gift card for partaking in the study.

### Data analysis

The focus group and individual interview audio recordings were transcribed verbatim and initially coded by two graduate students, followed by a thorough review by the three researchers with MPC credentials. An inductive reflexive thematic analysis was performed using Nvivo software. Braun and Clarke’s (2006, 2019) principles guided the analysis: (a) immerse oneself in the data for deep familiarization, (b) record initial ideas, comparisons, and reactions, (c) import transcripts and researcher notes into Nvivo software, (d) code the data and group codes into high-order themes, and work reflexively throughout the development and organization of themes to question and re-negotiate definitions and interpretations of the dataset. Through this process, the authors became very familiar with the data by re-reading the interviews multiple times. The graduate students worked in tandem to ensure they were using a similar framework during the initial coding. This was followed by multiple team meetings where all members of the analysis team reviewed and discussed each code, and initial groupings. With a deep understanding of the data, the latter was then coded and separated into higher order themes, sub-themes and categories. Through this reflexive process, the codes and themes were discussed and revised multiple times (Smith and McGannon, 2018), resulting in the current findings presented below.

## Results

### Context

The participants were exposed to various contexts in which they trained and competed. With constantly changing public health restrictions throughout the different waves of the COVID-19 pandemic across Canada, many athletes, coaches, and support staff members faced varying requirements. As one participant stated, “I think the pandemic affects everyone differently and the same people at different times. There are different situations, whether in training or locked out of training” (C#12). Further, it was noted by participants that Paralympic athletes may have experienced greater challenges due to their pronounced isolation, “I work also with some Paralympic sports and I think sometimes it has been harder during the pandemic because they are already usually a little bit more isolated socially and this social isolation was just amplified when they could not come to training” (S#10). Lastly, participants noted that athletes’ age may have had an impact on how athletes managed the pandemic, whereby younger athletes may have fared worse than older and more experienced athletes. Participant (C#9) said, “Specifically, the younger athletes I think are really confused off the field.”

### Theme 1: Consequences of COVID-19 for athletes

The first main theme, presented from both the athlete and coach/support staff perspective, and divided into two sub-themes—debilitative consequences of COVID-19 and facilitative consequences of COVID-19—which were, in turn, divided into categories (see Table 1). Some of the consequences of the pandemic overlap with certain factors from Theme 2. However, it is important to distinguish what *consequences resulted from* (Theme 1) the pandemic from what *contributed to* (Theme 2) athlete mental health during the pandemic. The first objective of the current study was to examine the interplay between the pandemic and mental health. This first theme addresses the first objective’s second question: what positively and negatively impacted athletes’ mental health during the pandemic.

**Table 1:** Theme 1: Consequences of COVID-19 for athletes.

Sub-theme	Category	Sub-category
Debilitative COVID-19 consequences	Low mood symptoms Anxiety and stress symptoms Maladaptive behaviors	
Facilitative COVID-19 consequences	Extra time	Time for life outside of sport Time to rest and recover Extra time to train
	Reconnection with sport Positive mental health	

#### Debilitative COVID-19 consequences

Participants discussed several debilitative consequences of the COVID-19 pandemic related to athlete mental health, including low mood, anxiety, and stress symptoms, and maladaptive behaviors.

##### Low mood symptoms

Participants noted several symptoms of low mood throughout the initial waves of the pandemic. For example, Participant A#14 stated, “Just a couple of months ago, I was sad, I was depressed, demotivated, struggling, not feeling good about myself, not feeling comfortable with where I was and it has been really tough and difficult.” Coaches and support staff noted several symptoms including lack of motivation and disengagement. As Participant C#4 described, “To not have access to this sport anymore, it’s 90% of what they do and their social life. It has been very difficult for some. One of our athletes did not even want to talk to us anymore.”

##### Anxiety and stress symptoms

In addition to symptoms of low mood, athletes expressed increased symptoms of anxiety and stress. Participant A#15 described, “I think I was definitely anxious at times. I would say mild anxiety. Just the way the world was going and not knowing what the next day might look.” Coaches and support staff also noticed an increase in stress and anxiety amongst their athletes. Participant C#8 summed it up, “Covid has made the training aspect or just everything a little bit more challenging and more stressful.”

##### Maladaptive behaviors

Some athletes described unhealthy or maladaptive behaviors because of the pandemic. For example, some participants described using cannabis consumption or overtraining behaviors as a means to cope, “I would just say smoking weed has been helpful. Probably to the point of addiction…” (A#2).

#### Facilitative COVID-19 consequences

Some athletes experienced facilitative consequences of the COVID-19 pandemic, favourably impacting their mental health. Participants noted that athletes benefited mostly by having extra time, by reconnecting with their motives for sport and by improving their mental health over time.

##### Extra time

Participants mentioned athletes benefited from the pandemic by having more time for life outside of sport as well as for rest and recovery, and extra time to train. This particular category helped address the second question of the second research objective of the current study: How did the athletes use the pandemic as an opportunity to work on their mental performance skills?

###### Time for life outside of sport

Many athletes mentioned that they benefited from the extra time to focus on aspects of their lives outside of sport, including time with family and friends, as well as pursuing educational goals. Participant A#8 said, “I started work on my Master’s through (School) because they have a pretty great program….” Athlete A#20 stated, “I reconnected with a lot of friends and family and was able to really get some relationships back. I think that was pretty valuable…a more balanced lifestyle because it was so crazy for us the year before.” Coaches and support staff discussed the importance of redirecting focus to other aspects of their lives and the pandemic provided athletes with this opportunity. For example, Participant S#7 stated, “Sport is good but there is more to life than sports. Losing a year is not the end of the world. We have to help people diversify their focus a bit.”

###### Time to rest and recover

A number of athletes noted that the extra time afforded due to the pandemic provided time to rest and recover from previous injuries. For example, Participant A#24 said, “After the Olympics got canceled, it hit me that nothing was going to happen this year and I could take a couple days off and let myself recover from overtraining injuries.” Coaches and support staff also mentioned the benefit of having extra time to rest and recover. Participant C#9 encouraged athletes to slow down and make use of the extra time, “I really tried to adapt and be positive, and get the athletes to use this as an opportunity to slow down.”

###### Extra time to train

Some athletes benefitted from the Tokyo 2020 postponement by being able to focus on training for an additional year. Participant A#22 described, “As I got the word that Tokyo was going to be pushed back a year, I was a little bit more joyful than other athletes and training partners. Just because I wasn’t fully confident in myself that I would be able to get back up there to meet the standards.” In addition to the extra year of training, some athletes mentioned that the extra time was useful to work on specific aspects of their sport, such as technical and mental skills. Participant A#15 described, “It was a good chance for me to kind of take a step back and focus on the technical stuff that I needed to improve on and I saw major gains because of that.”

##### Reconnection with sport

Some athletes noted that a positive consequence of the pandemic was having the opportunity to reconnect with their sport and reinvigorate their motives to participate. Participant A#21 noted their desire to inspire other athletes, “I just want to help motivate and inspire the next generation of athletes and especially athletes with disabilities and disabilities similar to mine.” Coaches and support staff observed athletes remembering why they love their sport. Participant S#11 summed it up by saying, “I have a couple of athletes saying that they are remembering why they fell in love with the sport in the first place. They are going through that process again while they are getting back all of their skills step by step, without that pressure of ‘oh you have to be ready in two weeks for this super important competition.”

##### Positive mental health

Some coaches and support staff shared that the pandemic may increase mental health in the future. Participant C#3 stated, “I think we are going to get the benefits of our mental health down the road, now that we kind of know each other or ourselves a little bit better. Maybe a little down the road, positive mental health can come out of this.”

### Theme 2: Factors impacting athlete mental health

The second main theme is divided into two sub-themes: Factors impeding athlete mental health and Factors facilitating athlete mental health. These factors were then divided into separate categories and sub-categories (see Table 2). This theme also addresses the first objective’s second question: what positively and negatively impacted athletes’ mental health during the pandemic.

**Table 2:** Theme 2: Factors impacting athlete mental health.

Sub-theme	Category	Sub-category
Factors impeding athlete mental health	Social and environmental	Isolation Stressors outside of sport Strained family relationships
	Psychological	Uncertainty of future Stigma Fear of virus Identity crisis
	Public health restrictions	Creation of disadvantages Disruption of routines and changes in plans
Factors facilitating athlete mental health	Social and environmental	Social support
	Psychological	Use of MPC & MHP Use of mental performance skills Autonomy Maintenance of a routine

#### Factors impeding athlete mental health

According to our participants, a number of factors contributed to challenges related to athlete mental health during the initial waves of the COVID-19 pandemic. These included sub-themes of social and environmental factors, psychological factors, and factors related to public health restrictions.

##### Social and environmental

The social and environmental factors that hindered athletes’ mental health included isolation, stressors outside of sport, strained family relationships, and communication fatigue.

###### Isolation

Athlete participants noted the immense challenges during the initial lockdowns and social isolation stemming from complete city-wide shutdowns. For example, Participant A#14 stated, “… during that quarantine, it has by far been the hardest thing to deal with because we are stuck inside, we are not socializing and we humans are social beings, we need that interaction.” Coaches and support staff described how being isolated seemed to create challenges for athletes, even if they were not members of a team sport. Participant C#4 stated, “It’s difficult because even if [sport] is an individual sport, it’s also a team sport. They could not see their teammates, they could not play against each other, and they did not have competitors!.”

###### Stressors outside of sport

Not surprisingly, athletes indicated stressors not related to their sport negatively affecting their mental health. In the initial stages of the pandemic, many socio-political and social justice events (e.g., Black Lives Matter) had an impact on athlete mental health. Participant A#6 noted, “Focusing a lot on Black Lives Matter movement and having discussions based on that” created additional challenges. Coaches and support staff also noticed how stressors outside of sport negatively contributed to athlete mental health. Participant S#5 shared how school had a negative impact on their athlete, “she could no longer handle not have good grades anymore and was very anxious about this. So I directed her to our regional multisport center for psychology services.”

###### Strained family relationships

Unique to athlete participants, while some may have appreciated the extra time spent at home with family, others noted that it causes additional challenges to their mental health, which was further amplified by the inability to use training as a coping mechanism. Participant A#18 stated, “I do not have the best relationship with my family. Having to sit at home all day meant that I had to be with them and spend time with them and argue with them all the time so me not being able to take out my stress with training was kind of rough for me.”

##### Psychological

The psychological factors that impeded athlete mental health pertained to the uncertainty of the future, stigma, fear of the virus and identity crisis.

###### Uncertainty of future

The pandemic resulted in many cancelations and uncertainty in the timing of events (i.e., when they would be reinstated). This caused additional challenges for athletes as noted by Participant A#6, “I feel like one thing that stands out to me is how much uncertainty there has been and all the changes and plans. Our team has attempted to put together so many training camps and they have all been cancelled and typically last minute.” Coaches and support staff also described how the uncertainty of the pandemic, particularly in the early stages, thwarted the well-being of athletes. For instance, Participant C#2 made reference to the “continued rumors around the Olympics this summer, and uncertainty.”

###### Stigma

Psychological resources to support mental health were available to athletes, however, the stigma associated with their use was perceived by some to be a barrier. Participant A#19 noted, “It’s almost like a weakness to be working with a mental health provider.”

###### Fear of virus

Coaches and support staff noted that the fear of contracting the virus had a negative impact on athlete mental health. Participant C#2 stated, “We have had a number of athletes who have had COVID-19, likely associated with the travel they had to do. I think there are potential lingering effects and levels of fear around getting it or having had it and not knowing how you got it.”

###### Identity crisis

Finally, coaches and support staff recognized that athletes seemed to be experiencing a form of identity crisis. That is, they observed athletes questioning their identity, their motivation, and their goals, which in turn created mental health challenges. Participant C#3 noted, “…COVID has made us check in with almost every aspect of who we are. Why we are training, what we are doing, and why we are doing it.”

##### Public health restrictions

The factors related to public health restrictions that impeded athlete mental health included the creation of disadvantages, the disruption of routines and the constant change of plans.

###### Creation of disadvantages

Many participants indicated that the varying public health restrictions created a perception of disadvantages between competitors. While some athletes in some training locations were able to train or compete, others in different areas and under different public health restrictions were not. This caused stress and strained participants’ well-being as Participant A#13 described, “I’d be on Instagram and see people on my team or people on other teams across the world still training and that was like, ‘oh my god, these people are training and I’m not training. They are getting an edge.’ That was not helpful.” Coaches and support staff, like athletes, expressed how the different public health restrictions created a perception of disadvantage. In the early stages of the pandemic, Canada (across all provinces and territories) had relatively strict public health restrictions compared to other countries (e.g., U.S.A., United Kingdom).

###### Disruption of routines and changes in plans

National team athletes are accustomed to following a relatively regimented training routine, therefore a significant disruption to that routine caused by varying restrictions created challenges to their mental health. This was noted by Participant A#13, “I think the biggest thing that was hindering my mental health with the pandemic was losing a sense of routine.” The dynamic public health restrictions across the country also led to frequent, and often last minute, change in plans. These continuous changes led to additional mental health challenges. Participant S#7 stated, “Things would change even on the same day and the athletes did not know if they were leaving for training camp, if they had a competition, and which ones would be maintained in what country.”

#### Factors facilitating athlete mental health

While the previous factors were perceived to hinder athlete mental health, a series of factors were described as facilitative. Participants noted several social/environmental and psychological factors that provided opportunities to enhance rather than threaten athletes’ mental health.

##### Social and environmental

A social and environmental factor deemed facilitative by athletes was social support.

###### Social support

Athletes noted the importance of having a strong support network, connecting with other athletes who could relate and understand their situation was also particularly helpful. As noted by Participant A#13, “Game Plan had a few athlete calls and I found those really help I…I found just hearing other athletes and their struggles was incredibly helpful for me to know that…we are not alone…we are all feeling the same things…to hear it from them directly was what I found really helpful.” Coaches and support staff noted that even if the teams could not be together in person, spending time together virtually and offering support was helpful for athletes’ mental health. Participant S#10 revealed, “The whole team would gather up for an hour [virtually] and chat about how the last two weeks was for them.” Coaches and support staff aimed to clearly communicate to athletes that they were there for support. Social support was often accessed through online platforms, allowing athletes to connect with others even when physical contact was not possible. Participant A#21 said, “I did not even know what Zoom was before Covid and every day, I’m on a Zoom call with someone now.” Athletes were able to use resources such as applications to reach registered mental health professionals, but also resources offered online by their own team and by national sport organizations to strengthen their mental health. Coaches and support staff identified that access to online resources facilitated athlete mental health via social support, as illustrated by Participant S#11, “Having Zoom has been a god send, and if this was 15 years ago, we would not really have been able to do any of the stuff that we did.”

##### Psychological

Several psychological factors were described as positive for athlete mental health and they included use of a Mental Performance Consultant (MPC) and/or a mental health practitioner (MHP), and use of mental performance skills. This particular category and its subsequent sub-categories help address the current study’s first question of the first research objective: How did athletes maintain their mental health during the pandemic and did their pre-pandemic mental health influence their responses to the pandemic and their ability to manage their mental health throughout, as well the first question of the second research objective: How did athletes’ pre-pandemic mental performance skills influence their responses to the pandemic and their ability to self-regulate throughout.

###### Use of MPC and MHP

Athletes noted that working with a MPC was beneficial to their overall mental health. Participant A#21 stated, “In hindsight, I am really thankful that I started working with [MPC] like 18 months before this happened.” Further, some athletes reached out to a MHP to more specifically work on their well-being, which was perceived as beneficial. Participant A#16 noted, “I feel like what helped the most was the psychologist, because he really knew what he was talking about. He really knew about the whole mental health aspect, especially how it took a big toll on all the athletes.” Coaches and support staff believed that athletes’ mental performance skills going into the pandemic influenced their experience. Participant S#10 shared, “It helped them and I know that in almost every team that I work with, there is an MPC that works with the athletes.”

###### Use of mental performance skills

In addition to the help of professionals during the pandemic, participants mentioned that mental performance skills they had previously acquired were helpful in maintaining their mental health. Participant A#13 said, “In the Spring, I got in the habit of journaling every day, meditating and listening to podcasts that were focused on mindfulness and performance, and I kept those routines even as things have kind of gotten somewhat back to normal.” Like athlete participants, coaches and support staff identified the use of mental skills as a facilitator of good mental health. Some emphasized the particular mental skill of resilience, as stated by Participant S#10, “[They] worked a lot on resilience abilities throughout the year. So how to become more resilient and get through the pandemic.” Coaches and support staff also recognized the importance of normalizing the use of an MPC and mental performance skill building, “There’s been a mental performance element built into our daily schedule, where like every day or every other day, there’s a 30-min time slot where every athlete is there. It’s not always conversation, sometimes it’s different neural training or activities, just to practice building habits. A lot of that is normalized.” (C2).

###### Autonomy

Coaches and support staff indicated that to facilitate athlete mental health, they would promote athlete autonomy amongst their team. For example, Participant C#6 would provide athletes with choices to help foster a sense of control and independence, “Personally, I liked to give them two choices. Similar to young kids, they had the impression that they had control and we made them accountable to do things well.”

###### Maintenance of a routine

Athletes mentioned the importance of maintaining a daily routine, often by waking up early, and having a set time for different activities. Athlete A#13 said, “the biggest thing that helped was re-establishing that routine.” Participants also expressed how maintaining their training level or trying new types of training was helpful in maintaining their mental health. As Participant A#25 explains, “I just tried to switch my mentality and be more like ‘I just want to be as fit as I can’ and do as many activities and different fitness challenges to become better, and be able to work on myself for the next year as well.” Coaches and support staff also mentioned that continued training and physical activity were instrumental. Participant S#10 revealed, “Even though you had a lot of sanitary rules, we were able to maintain some kind of regular training. I think it helped them too in regards to their mental health.”

### Theme 3: Coach and support staff perspective: an overview of their well-being and roles

The third main theme pertained to coach and support staff’s own well-being and their perceived roles throughout the initial waves of the pandemic. This theme is divided into two sub-themes: roles and well-being, (see Table 3) and addressed both the current study’s objectives in examining the role coaches and support staff played in supporting athletes’ mental health (Objective 1, Question 3) and mental performance (Objective 2, Question 3) during the pandemic.

**Table 3:** Theme 3: Coach and support staff perspective: an overview of their well-being and roles.

Sub-theme	Category
Roles	Promotion of well-being Preparation to return to competition Focus on coach-athlete relationship
Well-being	

#### Roles

Coaches and support staff noted that their roles during the initial waves of the pandemic included promoting well-being amongst their athletes and teams, preparing for the return to competition, and emphasizing the coach-athlete relationship.

##### Promotion of well-being

To promote well-being, participants fostered calmness and positivity, encouraged interests outside of sport, and took pressure off of athletes. For example, Participant C#8 shared, “I think my role in the whole process was a lot more about just trying to reinforce the positive mindset, or what positives could we take out of the situation.” With regards to exploring different interests outside of sport, Participant C#4 mentioned, “The objective was to get them to realize that their sport is not their entire life, that they are wholesome individuals, and that they have other things in life.” Finally, Participant C#12 revealed, “For me over the past year, I feel like I have been trying to take the pressure off the athletes.”

##### Preparation to return to competition

Coaches and support staff noted that their role in preparing for a return to competition was particularly important given the lack of international or major competitions leading up to the Tokyo Olympic and Paralymptic Games. Further, they noted how important it was for changes compared to previous Games and the additional security and sanitary precautions required.

##### Focus on coach-athlete relationship

Coaches in particular identified their role during this time to build their relationship with athletes. Participant C#4 described, “I have spent more time one-on-one with each athlete… I was able to discuss and get to know them better and they got to know me better as well.”

#### Well-being

Coaches and support staff had differing opinions regarding their well-being and ability to cope. For example, the pandemic provided some participants with the opportunity to increase their well-being by being at home with family and spending more time doing things outside of their sport. Participant C#9 revealed, “I have not really been home for more than a week for 20 years so for me that was a big adjustment. I really enjoyed this time at home, with my dog and in the mountains, so it’s been a real shift for me.” Other coaches and support staff noted the increase in stress and fatigue brought on by the pandemic, which depleted their well-being. Participant C#4 shared, “There was clearly a lot of stress and weight on our shoulders.”

## Discussion

The purpose of this research was to examine the interplay between the COVID-19 pandemic and the mental health and mental performance from the perspective of Canadian National team athletes, coaches, and support staff. Results from the present study show that the COVID-19 pandemic had both positive and negative consequences on National team athletes’ mental health. Negative consequences mentioned by the participants were mostly related to the athlete’s seclusion from their main life sphere, which has been previously tied to their identity, self-esteem, and social support network (Bissett et al., 2020; Freeman, 2021; Stephan and Brewer, 2007). The themes highlight a loss of routines for athletes and a difficulty in coping and readjusting their daily life leading to symptoms of low mood, anxiety and stress, and maladaptive coping behaviors. Recent research has tied the pandemic to consequences on athletic identity and, simultaneously, athlete mental health (Graupensperger et al., 2020), perhaps because some athletes were deeply engaged in their sport and had few opportunities to develop others. Conversely, some athletes reported that the forced interruption of access to their sports and the postponement of competitions felt like a much-needed pause. This break allowed them to rest and recover, gain more training time for improvement, and take a break from performance pressure. This finding of positive consequences on athletes’ mental health is surprising, as it is often assumed that Olympians and Paralympians thrive in their athletic lifestyle. These results suggest that some athletes might suffer from a lack of balance in their lives, not having sufficient time to rest, recover, and spend time outside of their sport. Results also indicate that while some athletes perceived negative impacts, they were able to find positive outcomes by taking advantage of factors within their control during these times. They used the opportunity to step back, focus on areas of improvement, and learn from adversity. This is encouraging and reinforce that resilience and coping are essential skills for athletes.

Regarding factors impacting athlete mental health, the results indicate that athletes are predominantly affected by elements related to their sport practice and environment. Being deprived of their sport practice not only disrupted their routine and performance goals but also created insecurity about their ability to perform in an uncertain future. Given the significant sacrifices and efforts invested by Olympic and Paralympic athletes toward their performance goals, it is unsurprising that the suspension of training and competitions in such an uncertain climate has caused distress for many athletes. Moreover, national team athletes, who spend significant amounts of time with their teammates and coaches, suffered from the isolation from this primary peer group. The results show that athletes affected by external stressors, such as academic difficulties or societal concerns, and those who report poor family relationships, perceived negative impacts on their mental health, particularly because they lacked access to sport as a coping mechanism. This indicates that when athletes are forced to take time away from their usual training routine and their team—whether due to injuries, offseason, or a global health crisis—they may face challenges outside of sport that can adversely affect their mental health and overall functioning. This underscores the importance of developing external support systems and investing in other life spheres, enabling athletes to rely on these resources. These results are in line with Kuettel and Larsen’s (2019) scoping review on the risk and protective factors for mental health in elite athletes, which found themes similar to ours and had elements related to the athlete’s sporting practice and personal environment (i.e., time away from sport due to injury, social support, uncertainty of career status, etc.). Therefore, there is a need to better equip athletes with a variety of coping skills and help them prepare for such eventualities.

Related to factors facilitating mental health, it seems like having the opportunity to keep meeting with their teammates, coaches and support staff through alternative online communication platforms helped athletes feel support and connected to one another. These social support networks were imperative for athletes; a concept that was reiterated by mental performance consultants who described social support as one of the main facilitating factors of an athlete’s ability to cope during the pandemic (Dithurbide et al., 2022). Athletes have a need to belong, feel supported and connected when facing adversity (DeFreese and Smith, 2014). These results are transferable to other situations where athletes may encounter injuries, non-qualifications, or mental health issues. Lessons learned during the pandemic can eventually be applied to these situations as well. The fact that coaches and support staff organized virtual meetings purposely as a means to provide support to athletes reinforces the key role they play in the wellbeing of athlete and in facilitating mental health support. Studies show that some coaches may feel reluctant to discuss mental health with athletes and that training coach mental health literacy is related to increase coach confidence in their ability to provide appropriate mental health support (Sankey et al., 2023). Results from the current study reinforce the benefit of helping coaches develop some basic skills related to mental health support. Going forward in a post-pandemic era, the ability to connect quickly and effectively through online platforms should be utilized to maintain a proactive approach to mental health and engage in early intervention.

The results also highlight the importance of providing specific mental health support resources for athletes. Opportunities for athletes to share their experiences with each other appear to be a valuable way to help them feel understood and less isolated in facing their challenges. Given that stigma surrounding mental health issues remains a significant barrier to seeking support (Cosh et al., 2023), offering opportunities for open discussions about mental health among athletes, coaches, and staff is a way to develop mental health literacy and reduce stigma. The current study indicates that support programs such as Game Plan for Canadian athletes have been beneficial. It is encouraging to see that the Canadian Game Plan program offers ongoing support to national athletes and coaches through various resources. The effectiveness of these programs would benefit from empirical study to better understand their mechanisms of action.

The current study, by providing the athletes’ perspective, confirms the importance of mental performance skills as coping strategies that are beneficial for mental health. Consistent with the perspectives of mental performance consultants (Dithurbide et al., 2022), the athletes’ viewpoint reveals that these skills, developed within the sports context, can be transferred to other settings and used for self-regulation in situations of adversity. Since COVID-19, there has been an increased focus on athletes’ mental health. As mentioned, this shift is positive as it has led to an increase in resources available to athletes since the pandemic. However, with limited resources in sport, this situation has also created competition for the allocation of these resources. In practice, sport psychology is a complex field. In North America, there is a distinction between mental health and mental performance, as well as between mental health practitioners and mental performance consultants (Durand-Bush and Van Slingerland, 2021). However, this distinction is often not well understood by the public, sport organizations, or even coaches, with some lumping all these issues under the umbrella of sport psychology. Without educational efforts, there is a risk of shifting the focus and investment predominantly toward mental health support for athletes, potentially favoring mental health professionals over mental performance consultants in working with athletes. However, both types of resources are equally important and fundamental to the well-being and success of athletes. The perspective of athletes and coaches in the current study demonstrates that by investing in athletes’ mental performance, athletes can develop skills that are beneficial to their mental health. Future studies are needed to confirm this link.

With respect to coaches and their perspective of the COVID-19 pandemic, our results suggest that these individuals were highly relied upon, which is a testament to their importance in an athlete’s life (Cassidy et al., 2023). Research done in the field of sport coaching advocates that coaching is a complex and interpersonal process that is composed of multiple different roles (Lyle, 2005; Thelwell et al., 2008). Coaches in our study claimed that they had to emphasize specific roles that went further than simply teaching their sport, such as promoting well-being, preparing their athletes to return to competition and focusing on developing a good relationship with their athletes. These results support recent literature on the reality and complexity of sport coaching, where coaches must shift between numerous responsibilities to ensure their athletes performance and well-being (Cassidy et al., 2023). Moreover, these results concur with a larger theme: the coach-athlete relationship. Coaching is often conceptualized as being a context where coaches work toward bringing about positive change in an athlete’s performance and well-being (Jowett, 2017). That being said, research has stipulated that this coach-athlete relationship could be seen as a key concept of coaching (Jowett, 2017).

The current study presents its own set of strengths and addresses current needs in the sport and exercise literature. A main strength of the current study pertains to its ties to the ecological framework and the importance it gives to an athlete’s support system (Gouttebarge et al., 2019). By interviewing athletes and members of their support staff, the present study attempted to gain a larger understanding of their realities during the COVID-19 pandemic. Another strength of the current study lies in its applicability to future situations. In studying athlete and support staff’s realities during the COVID-19 pandemic, the research team’s focus was on generalizable conclusions rather than pandemic specific conclusions. Hence, using the COVID-19 pandemic offered the study of a stressful situation, where athletes and support staff had to cope with disruption in their everyday lives.

Limitations to the current study include the varying levels of COVID-19 restrictions in Canada over the course of the study. Since our participants were Canadian, each lived in different provinces across the country, and in some cases, outside of the country. Hence, depending on where the participants lived and trained, they were subject to different set of restrictions. Considering the effect of one’s environment on their mental health and well-being, the COVID-19 pandemic situation in Canada could have affected our understanding of participant’s overall experience.

As seen through our results, when athletes face mental health challenges, they may engage in maladaptive coping strategies (e.g., substance abuse). Hence, it is important that sport organizations support athletes in their development of adaptive, healthy, and effective coping skills (Durand-Bush and Van Slingerland, 2021). Furthermore, sporting organizations should recognize the value of mental performance consultants and mental health practitioners on athletes’ development, as these experts teach several mental performance competencies that transcend every level of the ecological system (Bronfenbrenner, 1979).

## Concluding remarks

The current study revealed that athletes experienced a range of impacts during the COVID-19 pandemic—both positive and negative—shaped by various factors such as isolation, stigma, mental performance skills, and access to social support. These factors contributed to both good and poor mental health outcomes. The findings emphasized that mental health and mental performance are core elements of a culture of excellence in sport, and that they are interrelated and mutually reinforcing. Athletes who reported greater support and a deeper understanding of their reality appeared to have more cognitive and emotional capacity to invest in their sport and performance (Durand-Bush and Van Slingerland, 2021). This study also highlights the complexity of the coach–athlete relationship (Cassidy et al., 2023; Jowett, 2017), demonstrating the multiple roles coaches play and how these may shift during highly stressful periods, such as a pandemic, to prioritize and promote well-being. Sport organizations should prioritize the provision of resources and support—such as MPCs and mental health practitioners—to enhance both aspects of athletes’ psychological functioning, as recommended by Henriksen et al. (2020) and Liddle et al. (2017). As recent research underscores (Durand-Bush et al., 2023), fostering athletes who thrive in both sport and life requires a commitment to their overall well-being and a philosophy that puts the person before the performer. By doing so, sport organizations can optimize performance while safeguarding and promoting athletes’ mental health and quality of life—especially during times of extreme stress and adversity.

Based on these findings, sport leaders are encouraged to: (a) embed well-being and mental performance supports into daily training and organizational culture; (b) provide ongoing education for coaches and staff on adapting their roles to better support athletes’ psychological needs; and (c) establish formal mechanisms for checking in with athletes’ mental health regularly, particularly during times of systemic stress. This study contributes to the growing body of evidence that mental health and mental performance are inseparable in high-performance sport and should be addressed in an integrated manner across research, policy, and practice. Future studies are needed to examine the long-term effects of pandemic-related stressors on athletes’ mental health, identity, motivation, and performance, and to explore how sport environments can be proactively shaped to build resilience against future crises.

## Data Availability

The data presented in this article are not readily available due to institutional ethics approvals. Requests to access the data should be directed to Dr. Lori Dithurbide at: lori.dithurbide@dal.ca.
